# Gonadotropin Therapy for Mini-Puberty Induction in Male Infants With Hypogonadotropic Hypogonadism

**DOI:** 10.1210/clinem/dgae874

**Published:** 2024-12-14

**Authors:** Sophie Rhys-Evans, Francesco d’Aniello, Emma C Alexander, Ibrahim F Dinah, Sabine Heger, Anna Nordenstrom, Julia Rohayem, Sasha R Howard

**Affiliations:** Centre for Endocrinology, William Harvey Research Institute, Queen Mary University of London, EC1M 6BQ London, UK; Centre for Endocrinology, William Harvey Research Institute, Queen Mary University of London, EC1M 6BQ London, UK; School of Pediatrics, University of Rome Tor Vergata, 00133 Rome, Italy; Endocrinology and Diabetes Unit, Bambino Gesù Children's Hospital, IRCCS, 00165 Rome, Italy; Department of Paediatrics, St Mary's Hospital, Imperial College Healthcare NHS Trust, London W2 1NY, UK; Centre for Endocrinology, William Harvey Research Institute, Queen Mary University of London, EC1M 6BQ London, UK; Paediatric Endocrine Unit, Social Paediatric Centre AUF DER BULT, 30173 Hannover, Germany; Department of Women's and Children's Health, Karolinska Institutet, 171 77 Stockholm, Sweden; Paediatric Endocrinology, Ostschweizer Kinderspital, 9000 St Gallen, Switzerland; Centre for Endocrinology, William Harvey Research Institute, Queen Mary University of London, EC1M 6BQ London, UK; Department of Paediatric Endocrinology, Barts Health NHS Trust, London E1 1BB, UK

**Keywords:** gonadotropin treatment, gonadotropin deficiency, congenital hypogonadotropic hypogonadism, mini-puberty, fertility

## Abstract

**Context:**

Congenital hypogonadotropic hypogonadism (CHH) is defined as an isolated deficiency of gonadotropin hormones. Mini-puberty, a transient postnatal activation of the hypothalamic-pituitary-gonadal axis in healthy infants, provides a window of opportunity to diagnose and treat CHH. Currently, in male infants with CHH, testosterone is used to increase phallus size. However, gonadotropin replacement could additionally promote testicular descent and development, particularly relating to Sertoli cells. We conducted a systematic review of the effectiveness of gonadotropin therapy in stimulating mini-puberty related outcomes in male infants with CHH.

**Evidence Acquisition:**

In line with PRISMA guidelines, a systematic review of 11 databases was carried out (August 2023). Evidence quality was assessed using the Cochrane Risk of Bias for Non-Randomised Studies of Interventions tool. Protocol registered on PROSPERO (CRD42023453080).

**Evidence Synthesis:**

After a double-consensus screen of 767 abstracts and 66 full texts, 11 studies were included from 7 countries. A total of 71 male infants were enrolled, 12 with Kallmann syndrome. Median age at treatment initiation was 4.2 months (range, 0.25-57 months) and follow-up ranged from 3 to 10 years. Gonadotropin therapy was administered using continuous subcutaneous infusion (n = 35) or subcutaneous injection (n = 36). Due to treatment variability, modalities were combined for data synthesis. Gonadotropins induced a statistically significant increase in penile length and inhibin B concentration (*P* = .0007) and led to partial or full testicular descent in 73% (n = 62) of patients.

**Conclusion:**

This systematic review provides unique evidence supporting the efficacy of gonadotropins for induction of mini-puberty. However, the reliability and generalizability are limited due to disparate data and treatment modality variation.

Hypogonadotropic hypogonadism (HH) is a rare disorder underpinned by a disruption in the hypothalamic gonadotropin-releasing hormone (GnRH) neuronal regulatory network (1), which can be congenital (CHH) or acquired. This disruption results in a deficiency in the production, secretion, or action of GnRH (2), leading to reduced production of luteinizing hormone (LH) and follicle-stimulating hormone (FSH). Conditions of combined or multiple pituitary hormone deficiencies may also include impaired pituitary gonadotropin production, alongside adrenocorticotropin, thyroid-stimulating hormone, and growth hormone deficiencies.

It is estimated that the prevalence of CHH is between 1 in 4415 to 15 000 individuals, although high-quality recent data on the number of affected individuals internationally are limited. Males are diagnosed with this condition more commonly than females, at a reported ratio of 3.6 to 1 (3). The classic time for diagnosis of CHH is during adolescence, when individuals present to health-care providers with signs of delayed, absent, or arrested pubertal development. However, diagnosis in the early postnatal period is feasible, particularly in boys in whom “red flag’ clinical signs may be apparent shortly after birth and point toward this diagnosis. In particular, micropenis (a stretched penile length [SPL] < 2 SDs below the mean for age) and/or cryptorchidism (unilateral or bilateral undescended testis) are cardinal features that may alert a health-care provider to the need to investigate the underlying endocrine axis function (2, 4). These phenotypic signs are a result of hypothalamic-pituitary-gonadal (HPG) axis disruption in utero. CHH can also present with midline developmental anomalies, such as cleft lip and palate, ear and digit anomalies, sensorineural hearing loss, or renal agenesis (5). Additionally, hypoplasia of the olfactory bulbs results in the characteristic phenotype of anosmia, seen in Kallmann syndrome, which may be observed in infancy on brain magnetic resonance imaging scan by absence or hypoplasia of olfactory bulbs.

CHH can be confirmed biochemically in the first few weeks of life by the absence of the physiological postnatal surge in serum gonadotropins and consequently sex steroids that is observed in healthy infants during the period known as mini-puberty (6). Mini-puberty is a physiological event in early infancy characterized by the transient reactivation of the HPG axis, after the short period of axis quiescence immediately after birth. During mini-puberty an increase in pulsatile release of GnRH acts on the anterior pituitary to stimulate the secretion of LH and FSH. LH plays a central role in testicular Leydig cell stimulation, with a resultant rise in testosterone concentrations (mean peak serum testosterone at 3 months in a male infant of 4.02 nmol/L) (7). The effects of endogenous testosterone production in healthy males during mini-puberty are multiple, in promoting penile growth and scrotal development, but also for testicular descent into the scrotum (8). Human chorionic gonadotropin (hCG), which mimics the action of LH on its LH/choriogonadotropin receptor, can also be used to enhance Leydig cell function and intratesticular testosterone production (9). FSH stimulates Sertoli cell proliferation, which are a population of cells crucial to support spermatogenesis in postpubertal males. In healthy male infants this FSH-induced increase in Sertoli cell number is demonstrated by a measurable rise both in inhibin B (mean peak at 3 months in a male infant of 361 pg/mL) and antimüllerian hormone (AMH) secretion (median at 3 months in a male infant of 1046 pmol/L) (6, 7, 10-13). Inhibin B serves as a marker of Sertoli cell function, but also acts via negative feedback to regulate the central production of FSH (7), while AMH is secreted by immature Sertoli cells; low concentrations of both peptides suggest low Sertoli cell numbers (11). In health, the interplay between these hormones ensures coordinated development of the testicular structures; FSH optimizes germ cell support and proliferation, while LH and hCG-induced testosterone supports androgen-driven growth processes and testicular descent. Scheutz Henrikson and colleagues analyzed mini-pubertal markers of testis function in healthy boys at age 3 months, with follow-up at 18 to 20 years (n = 259), and identified serum testosterone concentration as a positive predictor of adult total sperm count (14). Mini-puberty in the first few months of life is therefore understood to be essential for the development of the male gonads, and can be viewed as a vital period of “priming” of the testes for future function. In healthy boys, gonadotropins then return to prepubertal levels by age 4 to 6 months, before rising again during normal puberty in adolescence (7, 8).

Male infants with CHH will therefore have low LH, FSH, testosterone, AMH, and inhibin B concentrations during mini-puberty (10, 15). Therefore mini-puberty offers a small window of opportunity both to diagnose and treat CHH early in childhood (16). Currently, treatment for male infants with micropenis involves testosterone or dihydrotestosterone to increase phallus size, while undescended testes are usually treated with surgical orchidopexy. However, exogenous testosterone will not promote physiological testicular development and orchidopexy, which will promote physiological development by bringing the testis to the scrotal position, can be technically challenging particularly for a small volume high-positioned testis.

Despite these clinical and biochemical indicators in infancy, the average age of initiation of meaningful treatment in males with CHH is 19 years (17). Delayed treatment is associated with negative lifelong repercussions, including reduced bone mineral density and psychological distress (15). Additionally, patients with a more severe CHH phenotype, with lower testes volumes, lower concentration of inhibin B, and unresolved cryptorchidism tend to respond less favorably to adult fertility treatment (18). One reason for this is the lack of Sertoli cell expansion during mini-puberty in individuals with CHH, resulting in reduced capacity for spermatogenesis after gonadotropin therapy to induce puberty or fertility (6, 19).

Combined gonadotropin replacement to recapitulate mini-puberty in male infants with CHH has the potential to both resolve cryptorchidism and to promote the expansion of Sertoli cells in the neonatal testis (20). A number of studies have shown that gonadotropin therapy, including recombinant human follicle-stimulating hormone (rhFSH) with either LH (rhLH) or the alternative hCG, are safe and efficacious treatments to induce puberty in adolescents and young adults (21). The administration of hormone replacement therapy reflects variation in the half-life of gonadotropins. LH has a short half-life, thus requiring either multiple daily administration or pulsatile delivery via continuous subcutaneous infusion pump, while hCG has a longer half-life, which allows for less frequent subcutaneous injections (9). rhLH or hCG can be given simultaneously with rhFSH in infants to replace mini-puberty, as before age 4 to 5 years Sertoli cells do not express the androgen receptor and thus—unlike in adolescents—administration of rhFSH with rhLH or hCG will lead to Sertoli cell proliferation without differentiation. While case studies and series of the use of these medications to emulate the physiological activation of the HPG axis in mini-puberty in male infants with CHH have been published, no systematic review of this literature on this emerging area of CHH treatment is available, and the availability and clinical use of these therapies internationally remains limited.

This systematic review aims to enhance the current literature base by formally evaluating the published evidence regarding the efficacy of gonadotropin therapy for inducing mini-puberty in male infants, using clinical and biochemical outcome measures.

## Methods

### Registration and Search Strategy

This review was registered on PROSPERO (CRD42023453080). The conduct of this review complied with the Declaration of Helsinki. We searched CENTRAL, Cumulative Index to Nursing & Allied Health (CINAHL), ClinicalTrials.gov, Embase, Google Scholar, MEDLINE, PsycINFO, PubMed, Scopus, Web of Science, and the World Health Organization (WHO), from inception until August 2023. Our search terms combined (hypogonadotropic hypogonadism [and derivatives] AND gonadotropins [and derivatives] AND infants [and derivatives]). The full search strategy can be found in the supplementary material (22).

### Eligibility Criteria

The population included male infants up to age 2 years diagnosed with CHH by clinical and biochemical criteria, who received an intervention of gonadotropin or GnRH therapy for the purpose of stimulating mini-puberty. Treatment courses of 2 or more doses were included. Comparators were any or none. The assessed primary outcome was efficacy, which was evaluated using testicular position, testicular volume (mL), SPL (mm), penile girth (mm), LH (IU/L), FSH (IU/L), testosterone (nmol/L), inhibin B (pg/mL), and AMH (pmol/L). Secondary outcomes were long-term follow-up (defined as >1 year after treatment cessation) of primary outcomes, fertility outcomes (if applicable), and safety profile (including adverse or reportable outcomes). All studies with human participants were eligible for inclusion.

Exclusion criteria were as follows: no gonadotropins or GnRH administered; no mini-pubertal outcomes included: no primary results; animal study; studies in which all participants were older than 2 years at initial treatment; full text not available; combined pituitary hormone deficiency without low gonadotropins; testosterone therapy monotherapy given; primary gonadal failure causing hypogonadism; not published in English/no translation available; outcomes not quantified; female participants; critical level of bias; and duplicates.

### Data Extraction and Quality Appraisal

The retrieved titles and abstracts, and shortlisted full texts, were screened by 2 independent researchers (SRE, FdA, IFD, SRH) using the Covidence platform (www.covidence.org). Any discrepancies were resolved by discussion. Data extraction was performed by one researcher and checked by a second.

Quality appraisal was performed by 2 independent researchers (SRE, SRH) and discrepancies were resolved by discussion or by the senior author (SRH). We used the Cochrane Risk of Bias for Non-Randomized Studies of Interventions (ROBINS-I) tool for nonrandomized studies. The threshold for an overall score of “serious” was if the risk of bias was “serious” in 3 or more domains, as agreed by consensus. We anticipated a small number of included studies in this systematic review (based on prior preliminary searches). Therefore, we did not adopt exclusion criteria based on calculated risk of bias but have outlined the risk of bias for all studies.

### Data Synthesis

Meta-analyses could not be carried out due to heterogeneity of interventions, settings, study designs, and outcome measures. A narrative synthesis was therefore performed, reporting the study design and descriptive characteristics of the participants in the included studies. Data synthesis was performed on available individual patient data. The discussion was used to report the limitations of the synthesis, given the anticipated heterogeneity of studies and outcomes to be included.

Univariate comparison was performed between pretreatment and posttreatment outcome variables. Categorical data were described as numbers and percentages. All continuous data were tested for normality using the Shapiro-Wilk test. Parametric data were described using mean and SD, and an unpaired 2-tailed *t* test was applied for univariable comparison. Nonparametric data were described using median and interquartile range and analyzed using a Mann-Whitney test. Where enough individual participant data were available, a paired *t* test was applied to matched pretreatment and posttreatment individual patient data values. Median and interquartile range (IQR) were only used to represent the cohorts age, due to limited individual data availability in two studies (23, 24). An adjusted *P* value was calculated from individual data points and statistical significance was taken at *P* less than .0014, as the threshold for statistical significance post Bonferroni adjustment. When small sample sizes allowed, subgroup analyses were conducted for pump and injection groups, on pretreatment and posttreatment outcomes. All plots included individual data points to allow *P* values and data synthesis to be interpreted in the context of the small study sample size. Data were processed in Microsoft Excel 2024, and comparative analyses were performed in GraphPad, Prism version 10.2.1.

## Results

**Figure 1.**
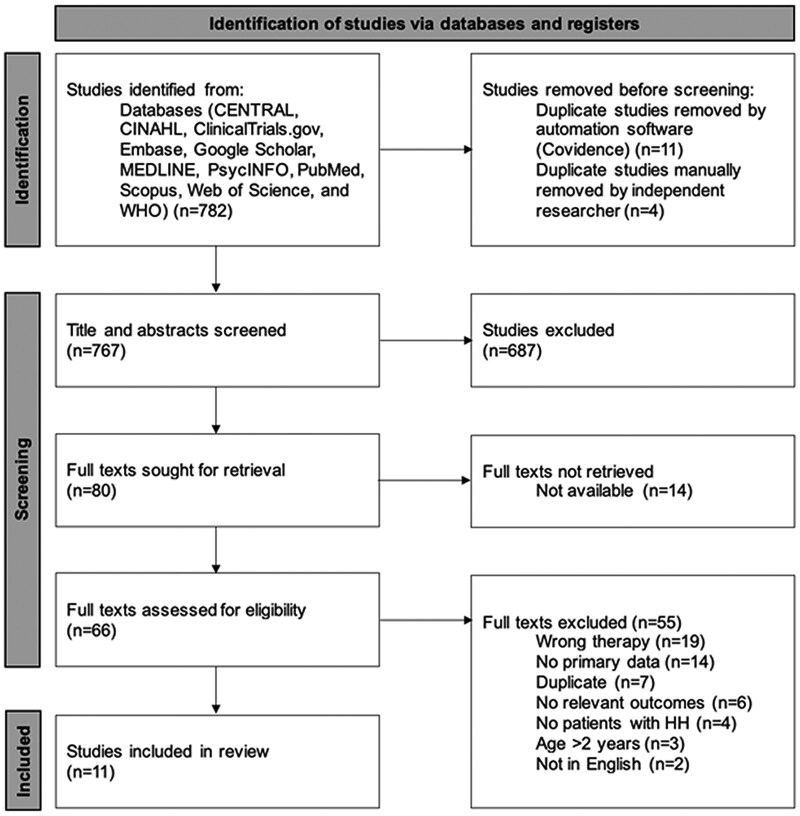
PRISMA 2020 flow diagram for new systematic reviews that included searches of databases. From: Page MJ, McKenzie JE, Bossuyt PM, Boutrin I, Hoffman TC, Mulrow CD, et al The PRISMA 2020 statement: an updated guideline for reporting systematic reviews. BMJ 2021: 372:n71. doi: 10.1136/bmj.n71. For more information, visit: http://www.prisma-statement.org/. CINAHL, Cumulative Index to Nursing & Allied Health; WHO, World Health Organization.

The Preferred Reporting Items for Systematic Reviews and Meta-Analyses (PRISMA) flow diagram in Fig. 1 illustrates the study screening process. Our search retrieved a total of 782 studies, and 11 studies met the inclusion criteria (all were observational studies). These described 71 patients across 7 countries. A summary of the treatment received in the included studies can be found in Table 1.

**Table 1.** Patient characteristics and dosing regimens of the 11 included studies

Citation	Study design	CHH cases, n	Age at treatment initiation, mo	Treatment method	Gonadotropin/ GnRH regimen (all doses in IU, except GnRH in mcg)	Treatment length, mo	Gonadotropin/ GnRH total dose (all doses in IU, except GnRH in mcg)
Alvarez Casano 2019 (25)	Case series	2	0.5-1.5	Injection	hCG 250-500 × 2/wk + FSH 37.5-75 × 3/wk*^a^*	4.5-6.5	hCG 9000-23000 + FSH 2025-5175
Stuckey 2023 (26)	Case report	1	12	Pump	GnRH 50-200/d	2	GnRH 7604-9885
Main 2002 (27)	Case report	1	7.9	Injection	LH 20-40 × 2/wk + FSH 21.3 × 2/wk*^b^*	3.4-4.9	LH 816 + FSH 834.96
Bougneres 2008 (28)	Case series	2	2-5	Pump	LH 50-56/d + FSH 67-125/d	4.5-7	LH 6664-9800 + FSH 7973-24 500
Sarfati 2015 (29)	Case series	1	1	Pump	LH 75/d + FSH 75/d	6	LH 13687.5 + FSH 12 687.5
Lambert 2016 (30)	Case series	8	0.25-11	Pump	LH 50/d + FSH 75-150/d*^c^*	5-6.5	LH 7604-9885 + FSH 22 813-29 656
Stoupa 2017 (31)	Cohort study	5	3-5.5	Pump	LH 75-150/day + FSH 75/d	3-6	LH 13 688-22 813 + FSH 6844-13 688
Papadimitriou 2019 (23)	Cohort study	10	2.28-9.36	Injection	LH 75/day + FSH 150/d	3	LH 6750 + FSH 13 500
Sharma 2021 (32)	Cohort study	1	1.5	Injection	hMG 25 × 3/wk + hCG 250 × 2/wk	3-5	hMG 600-900 + hCG 6000
Avril 2023 (24)	Cohort study	18	0.75-11.5	Pump	LH 75/day + FSH 75/d	6	LH 13687.5 + FSH 13687.5
		17	1-57	Injection	FSH 25 × 3/wk + hCG 260 × 2/wk	3	FSH 900 + hCG 6240
Kohva 2019 (33)	Cohort study	5	0.7-4.2	Injection	FSH 7.5-16.7 × 2-3/wk*^d^*	3-4.5	FSH 199-601

Abbreviations: CHH, congenital hypogonadotropic hypogonadism; FSH, follicle-stimulating hormone; GnRH, gonadotropin-releasing hormone; hCG, human chorionic gonadotropin; hMG, human menopausal gonadotropin; LH, luteinizing hormone.

^*a*^Growth hormone given in n = 1 for last 1.5 months of gonadotropin therapy.

^*b*^Testosterone 1 mg/day given in n = 1 for last 1.5 months, alongside FSH only.

^*c*^Classic replacement therapy (growth hormone, L-thyroxine, hydrocortisone) in n = 8 for therapy duration.

^*d*^Testosterone 25 mg × 1/month in n = 5 for 3 months.

### Summary of Patient Cohorts and Treatment

At treatment initiation, the median age of the 71 male infants was 4.2 months (from median and individual patient data, IQR 0.25-57 months). All cohorts included boys with idiopathic (I)HH or CHH, as per inclusion criteria. The most common diagnosis was IHH (n = 26, 37%), while 12 boys were explicitly described as having Kallmann syndrome, and 15 boys exhibited another form of syndromic CHH (Supplementary Table S1) (22). All boys had micropenis (100%), and 68% also had bilateral cryptorchidism (Supplementary Table S2) (22).

Gonadotropins (or GnRH in one study) were administered using continuous subcutaneous infusion (or “pump’) in 6 of the studies to 35 patients. Avril and colleagues (24) analyzed 2 different treatment regimes, 1 administered via pump, the other via injection, to 2 separate cohorts (see Table 1). For the following descriptive data in this paragraph, these 2 cohorts are considered as separate studies, thereby totaling 12 studies. Across the studies, rhFSH was administered in 83% (n = 10), rhLH in 58% (n = 7), hCG in 25% (n = 3), and GnRH and human menopausal gonadotropin (hMG) in 1 study each (8%). Testosterone was used as combination therapy in 2 studies, and data points that could have been skewed by this exogenous testosterone (ie, serum testosterone concentrations) were excluded as necessary. Treatment doses, frequency, and duration were highly variable across all studies (see Table 1). The treatment length varied from 2 to 7 months, with total dose administered ranging widely: rhFSH from 199 to 29 656 IU, rhLH from 816 to 22 813 IU, hCG from 6000 to 23 000 IU, hMG from 600 to 900 IU, and GnRH from 7604 to 9885 mcg. Posttreatment values, when stated, were measured within 6 months of the final gonadotropin dose. Follow-up was available for one study at a mean age of 11 (±1.5) years (33).

### Quality Appraisal

All nonrandomized studies underwent quality appraisal according to ROBINS-I guidelines (34). Table 2 illustrates the scoring of bias for individual domains and the overall consensus score. Most of the studies demonstrated “moderate” bias across the domains, and overall, none were comparable to well-performed randomized trials. However, one study was highlighted as having serious problems with classification bias and missing data, which implicated a reporting bias (26). Intervention bias was also identified when testosterone supplementation was given during the evaluated treatment period (27, 31, 33), or when different treatment regimens were administered and the results combined (24). Where affected outcomes were measured, these data was excluded from analysis. No studies had a “critical” level of bias to warrant being excluded from the synthesis.

**Table 2.** Quality appraisal of all included studies as per Risk of Bias for Non-Randomized Studies of Interventions (ROBINS-I), carried out by 2 independent researchers (20)

Citation	Bias due to confounding	Bias in selection of participants into study	Bias in classification of interventions	Bias due to deviations from intended interventions	Bias due to missing data	Bias in measurement of outcomes	Bias in selection of reported result	Overall consensus
Alvarez 2019 (25)	Moderate	Moderate	Moderate	Moderate	Moderate	Moderate	Moderate	Moderate
Stuckey 2023 (26)	Moderate	Moderate	Serious	Moderate	Serious	Moderate	Serious	Serious
Main 2002 (27)	Moderate	Moderate	Moderate	Serious	Moderate	Moderate	Moderate	Moderate
Bougneres 2008 (28)	Moderate	Moderate	Moderate	Moderate	Low	Moderate	Moderate	Moderate
Sarfati 2015 (29)	Moderate	Moderate	Moderate	Moderate	Serious	Moderate	Moderate	Moderate
Lambert 2016 (30)	Moderate	Low	Moderate	Moderate	Low	Moderate	Moderate	Moderate
Stoupa 2017 (31)	Moderate	Moderate	Moderate	Serious	Moderate	Moderate	Serious	Moderate
Papadimitriou 2019 (23)	Low	Low	Moderate	Moderate	Serious	Moderate	Moderate	Moderate
Sharma 2021 (32)	Moderate	Serious	Serious	Moderate	Low	Moderate	Moderate	Moderate
Avril 2023 (24)	Low	Moderate	Moderate	Serious	Moderate	Moderate	Serious	Moderate
Kohva 2019 (33)	Low	Moderate	Moderate	Serious	Moderate	Moderate	Moderate	Moderate

Any discrepancies were resolved by discussion. “Low” (no shade) indicates that this domain is comparable to a well-performed randomized trial, “moderate” (light shade) indicates that this study provides sound evidence for a nonrandomized study but cannot be considered comparable to a well-performed randomized trial, and “serious” (dark shade) indicates that this study has some important problems. The overall consensus indicates the judgment of this study across all domains.

### Clinical Outcomes

**Figure 2.**
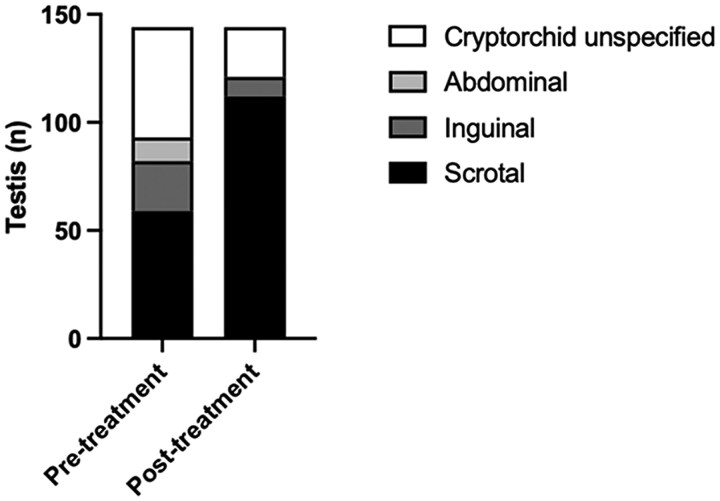
Change in testicular position before and after treatment with gonadotropins. Testis reported as high-scrotal are included in the inguinal group. No descent is defined as abdominal, partial descent is defined as inguinal, and full descent is defined as scrotal. Total sample size of testis is 144.

Testicular position was recorded in 10 out of the 11 studies. Due to some patients having unilateral cryptorchidism, testicular position has been described for 2 separate testis for each individual patient (n = 144). Out of the possible 6 studies that recorded 85 cryptorchid testis at treatment initiation, gonadotropin treatment was found to stimulate partial or full testicular descent in 73% (n = 62) of testes (Fig. 2, Supplementary Table S3) (22).

Table 3 outlines the pretreatment and posttreatment continuous data synthesis from 9 studies that provided individual patient data values. Due to missing data, 2 studies have been excluded from data synthesis (23, 24). There were not sufficient data available to carry out a data synthesis of penile girth.

**Table 3.** Continuous data synthesis of clinical and hormonal outcomes before and after treatment in 9 studies, for which raw data values were available (19, 21-24, 26, 27, 33, 34)

Outcome	Pretreatment (mean ± SD)	Posttreatment (mean ± SD)	*P*	Adjusted *P^a^*
Testicular volume, mL	0.35 ± 0.30 (n = 12)	1.29 ± 0.81 (n = 17)	.0007	.0049
Stretched penile length, mm	17.71 ± 5.37 (n = 24)	36.54 ± 11.36 (n = 24)	<.0001	.0007
LH, IU/L*^b^*	0.05 (0.11) (n = 17)	2.45 (3.38) (n = 12)	<.0001	.0007
FSH, IU/L	0.30 ± 0.31 (n = 18)	17.83 ± 18.37 (n = 15)	.0003	.0021
Testosterone, nmol/L	0.14 ± 0.07 (n = 25)	7.73 ± 8.89 (n = 14)	.0001	.0007
Inhibin B, pg/mL	71.42 ± 44.64 (n = 18)	275.00 ± 166.50 (n = 17)	<.0001	.0007
AMH, pmol/L	694.50 ± 579.90 (n = 11)	1067.00 ± 852.20 (n = 10)	.2516	.8685

Data pass normality testing using the Shapiro-Wilk test and are represented by mean and SD. Sample size is shown in parentheses for each data synthesis. *P* values generated using an unpaired *t* test. Statistical significance is taken at *P* less than .0014.

Abbreviations: AMH, antimüllerian hormone; FSH, follicle-stimulating hormone; LH, luteinizing hormone.

^*a*^Post Bonferroni adjustment.

^*b*^Nonparametric data shown as median (interquartile range).

**Figure 3.**
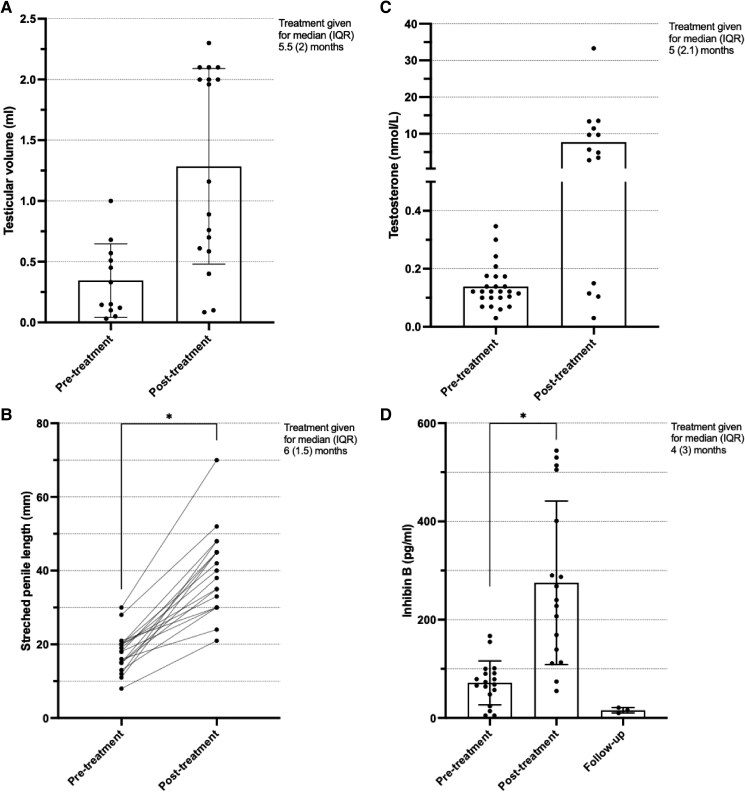
(A) Testicular volume (mL) (individual patient values with mean and SD) before and after gonadotropin therapy. Sample size in pretreatment group was 12 and posttreatment group was 17. (B) Stretched penile length (mm) (individual patient values) before and after treatment gonadotropin therapy. Sample size in pretreatment and posttreatment groups was 18. (C) Testosterone (nmol/L) (individual patient values and mean) before and after gonadotropin therapy. Sample size in pretreatment group was 25 and posttreatment group was 14. Y axis split at 0.5 to illustrate the lower range of pretreatment data. (D) Inhibin B (pg/mL) (individual patient data with mean and SD) before and after gonadotropin therapy, and at follow-up in one study (33). Sample size in pretreatment group was 17, posttreatment group was 15, and follow-up was 3. IQR, interquartile range. *P = .0007.

Testicular volume was measured in 10 studies, 9 using ultrasound, with 8 identifying an increase following gonadotropin treatment. Across the studies with independent patient data available, testicular volume increased from 0.35 (±0.30, n = 12) to 1.29 mL (±0.81, n = 17; *P* = .049) (Fig. 3A).

SPL was the most well-documented outcome across all clinical and biochemical parameters, measured by 10 studies that all demonstrated an increase in length following gonadotropin therapy, which reached statistical significance on data synthesis (see Table 3). Due to less data heterogeneity, it was possible for this parameter to match pretreatment and posttreatment values for individual patients, although sample size was reduced from 24 to 18 individuals. SPL increased from 18 (±5.5) to 40 mm (±11.6; *P* = .0007) (Fig. 3B).

### Biochemical Outcomes

Eight studies (72%) included data on testosterone serum concentration, either measured in nanograms per milliliter or nanomoles per liter. SI units are adopted for this paper, and values have been converted where necessary. Within these 8 studies, pretreatment testosterone concentrations increased from 0.14 (±0.07, n = 25) to 7.73 nmol/L (±8.89, n = 14) (Fig. 3C). Of note, the concentrations remained undetectable in Main and colleagues’ case report (27), and Álvarez Casaño and López Siguero (n = 2) (25) identified a transient improvement (peak values 22.9 and 50.5 nmol/L) before concentrations rapidly returned to undetectable during and 1 month after gonadotropin therapy.

Eight studies (72%) measured inhibin B as a hormonal indicator of Sertoli cell function following gonadotropin treatment, demonstrating a statistically significant increase from baseline at treatment cessation, from 71.43 (±44.6, n = 18) to 275 pg/mL (±166.5, n = 17; *P* = .0007) (Fig. 3D). However, one study found this increase to be transient, albeit following relatively modest doses of rhFSH (16.6 to 33.2 IU per week for 3-4.5 months, with the limited increase in patient's serum FSH concentrations suggesting insufficient dosing) (33). Due to the lack of consensus in the literature, the dose used in this study was guided by results from the authors’ previous work on prepubertal boys in 1997 (35).

Follow-up data were limited across all studies, apart from in this same patient group (n = 3), who not only demonstrated a return to baseline inhibin B concentrations at treatment cessation, but a reduction in inhibin B concentrations to below pretreatment levels (15.7 ± 5.5) at a mean age of 11 (±1.5) years’ follow-up (Supplementary Table S4) (22).

AMH was measured by a smaller number of studies (45%), where an improvement in posttreatment values was noted by all (from 695 ± 580 to 1067 ± 852 pmol/L) but this did not reach statistical significance.

### Direct Comparison of Gonadotropin Administration Methods

**Figure 4.**
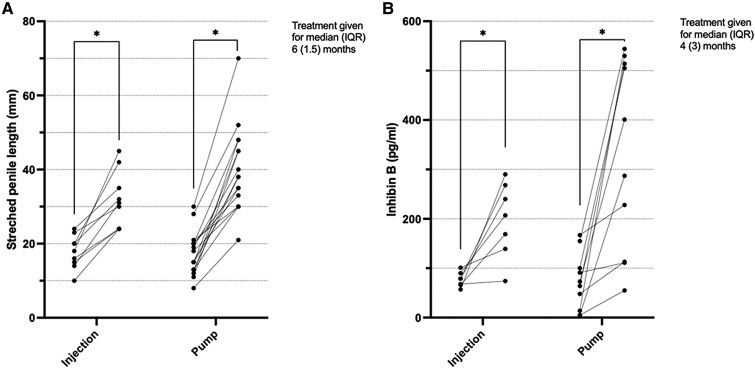
Individual patient values of (A) stretched penile length (mm) and (B) inhibin B (pg/mL) before and after treatment with treatment administered via a pump or via subcutaneous injection. Sample size in A, injection group was 9 and pump group was 16, and B, injection group was 7 and pump group was 14. IQR, interquartile range. *P = .0007.

Half of the studies that measured change in SPL and inhibin B concentrations did so following pump infusion (n = 6), while the other half administered gonadotropins via subcutaneous injections. There was large variability between doses and length of therapeutic regimens (see Table 1). This analysis considers the data from the pump cohort of Avril et al (24) as separate from those from the same study who received injections, as each received different therapy, duration, and dose. A statistically significant increase in SPL was seen after treatment with both methods, with neither superior to the other (Fig. 4A). A significant increase in serum inhibin B concentrations after treatment was noted with both methods, again with no statistically significant difference between the two modalities (Fig. 4B).

### Adverse Effects

Four studies (36%) reported the presence or absence of adverse events during the evaluated treatment period (see Supplementary Table S4) (22). One study (n = 1) reported the presence of minor adverse events, describing recurrent otitis media, initial sleep disturbances, intermittent nausea, and an increase in body hair and pigmentation (27). This group also reported one episode of local rash at injection site. This patient had a diagnosis of IHH, with normal pituitary axes, and was born to consanguineous Turkish parents. No other adverse effects were reported in the other 3 studies that investigated for such (23, 28, 30). Both treatment methods appear to be well tolerated across all studies.

### Follow-up

Seven studies (63%) provided follow-up data that ranged from 3 to 10 years after gonadotropin treatment cessation (see Supplementary Table S4) (22). Two studies reported testicular ascent in 3 patients (1 bilateral, 2 unilateral) within a year of treatment cessation, which were all successfully corrected with orchidopexy (23, 30). One year after treatment cessation, Sarfati et al (29) noted a reduction in testicular volume from 2.3 mL to 0.8 mL, whereas the study by Álvarez Casaño and López Siguero (25) reported testosterone concentrations returning to undetectable levels despite penis size and testicular volume remaining constant. Importantly, there were no reports of any infant followed up to an age when pubertal development would be expected.

## Discussion

It has been hypothesized that combined gonadotropin therapy to induce mini-puberty in male infants with CHH could lead to improved long-term outcomes for testicular development and spermatogenesis, as compared to traditional therapy with testosterone. The current evidence base has not yet been able to draw clear conclusions on the clinical and hormonal effect of this early postnatal replacement therapy, nor on long-term outcomes for fertility. This systematic review therefore represents the most comprehensive synthesis of clinical and hormonal mini-puberty outcomes in infants with CHH treated with gonadotropins or GnRH, including testicular descent, testicular volume, SPL, testosterone, inhibin B, and AMH concentrations. Within the moderate sample size there is substantial study heterogeneity in the selection, dosing, and duration of treatment across all studies, alongside potential bias at the study level. However, we have demonstrated that there is evidence for the efficacy of gonadotropin therapy, with more than 70% of studies reporting an increase in testicular descent and volume, SPL, testosterone, and inhibin B concentrations following treatment.

In an effort to increase sample size, we performed our own individual patient data synthesis and found that gonadotropin therapy during mini-puberty significantly increased SPL and inhibin B in the collated CHH cohort. Pitteloud and colleagues (18) identified that in adult men, a baseline inhibin B greater than 60 pg/mL (n = 76) was a favorable predictor for achieving good testicular volumes and thus increasing spermatogenesis in response to adult fertility induction. This concordant relationship between these 2 parameters is supported by our data synthesis, with 6 of the 8 studies that reported testicular volume and inhibin B serum concentration demonstrating an increase in both parameters in response to infantile gonadotropin therapy. It is possible that an increase in these positive predictors of fertility outcomes might suggest that gonadotropin replacement of mini-puberty may lead to improvements in long-term fertility outcomes for infants with CHH.

It would thus be anticipated that these patients will have higher inhibin B (and AMH) concentrations prior to entering puberty (23, 28, 32). However, the one study to report follow-up of 3 patients at a mean age of 11 years found that prepubertal inhibin B concentrations had returned to pre–gonadotropin treatment concentrations (33). It must be noted that these infants were treated with the lowest rhFSH dose across the studies, and they did not achieve target serum concentrations of FSH during mini-puberty. Again, more follow-up data are needed, and we anticipate the future publication of adolescent inhibin B concentrations from patients treated with combination gonadotropin therapy during mini-puberty (23, 24).

With regard to stimulation of testicular descent during mini-puberty, it is important to consider the need to mitigate surgical interventions such as orchidopexy. We found that gonadotropin therapy during mini-puberty stimulated partial or full testicular descent in the majority of patients (73%). While the optimal treatment duration, selection, or dosage cannot be derived from this synthesis due to substantial study heterogeneity, Avril et al (24) reported that the risk of cryptorchidism decreased significantly over time during treatment (odds ratio = 0.97 [0.96-0.99] per day; *P* < .0001). This risk reduction was similar between injection and pump treatment groups. In contrast, Kohva and colleagues (33) reported testicular reascent, despite gonadotropin treatment, with 5 boys requiring orchidopexy; however, these patients received relatively low-dose rhFSH administered as a monotherapy. Together the summative data show that in those with undescended testes prior to gonadotropin therapy, more than half achieved testicular descent preventing the need for orchidopexy. Those studies that report testicular descent also demonstrate an increase in testicular volume (24, 30-32). It is possible that in infants requiring orchidopexy after gonadotropin treatment, the augmented testicular volume and longer spermatic cord post therapy could potentially facilitate the procedure and mitigate surgical risks, thereby improving postoperative outcomes (36). Gonadotropin therapy could be considered as a first-line treatment option in this patient cohort with CHH and cryptorchidism, but long-term follow-up data with orchidopexy outcomes are needed to support this recommendation.

In terms of treatment safety, infants showed no serious adverse effects, indicating that gonadotropin replacement therapy has a favorable safety profile. Some conclusions about the safety profile of gonadotropin therapy can reasonably be extrapolated from the large number of studies conducted in adolescents (21). However, due to the small number of infants treated during mini-puberty, safety considerations remain paramount. Moreover, further research is needed to evaluate the safety profile of gonadotropin therapy during mini-puberty compared to in adolescence or adulthood. It would also be useful to directly compare side-effect profiles of gonadotropin therapy to those of alternative treatments in mini-puberty such as exogenous testosterone.

A major barrier to replacement of mini-puberty in patients with CHH is lack of timely diagnosis. Micropenis and cryptorchidism are apparent shortly after birth and should be identified at postnatal checks, ideally triggering further investigation to confirm the absence of the expected gonadotropin surge in the first few months of life in infants with CHH (15). However, pick-up rates of boys with CHH in the postnatal period remain disappointing. Contributing to this may be a lack of awareness of the benefits of earlier treatment initiation, and our aim is that this systematic review may contribute to reducing this issue. A proactive approach and development of a prediction tool to improve detection of infant boys with CHH, as proposed by Swee and Quinton (15), is an important area for future research.

To support this endeavor, we have recently developed a standardized clinical practice guideline for diagnosing and treating male infants with CHH (8), as well as an accessible electronic registry for longitudinal data collection of patients with HH of all ages and etiologies, named I-HH (fourth module in the SDM registries series https://sdmregistries.org/) (37). The latter can aid the collection of high-quality follow-up data, during puberty and on into reproductive life and offers valuable insights into spermatogenesis and fertility outcomes. Furthermore, the potentially beneficial effect of postnatal treatment on physical and psychosocial well-being is an important factor to consider within longitudinal data collection, particularly when compared to postpubertal gonadotropin therapy. Ideally, randomized controlled clinical trials of gonadotropin therapy during mini-puberty would clarify the optimal dosing regimen and evaluate long-term outcomes. This approach is also needed to differentiate the effect of replacement of mini-puberty from that of pubertal intervention for fertility and non-reproductive sequelae and potential confounding between these two important developmental stages, although it is still unable to account for the absence of elements of physiological mini-puberty and adolescent puberty that cannot be fully replaced by exogenous gonadotropins, such as the potential extra-hypothalamic effects of GnRH on the developing brain. However, with a rare disease such as this, to achieve this there must be a coordinated collaboration across international centers. Such a collaboration across multiple centers and countries would support the development of a common protocol through prospective studies to determine best practice in the treatment of infant boys with HH.

## Limitations

There are several limitations to this systematic review, which stem from the rare study population and thus modest sample size. As anticipated, a small number of studies were included from the screening process, all of which had a relatively small number of participants, leading to imprecise estimates. However, the included studies directly addressed our review question and provide data that are therefore highly relevant and applicable.

All studies were observational and performed on a small number of patients in a clinical setting. As a result, most assessors used different treatment regimens for each patient, and were aware of the intervention status, changing the treatment dose accordingly. This introduces intervention bias and reduces the applicability of our findings. However, this approach reflects the unique patient cohort and is understandable given the clinical environment. We needed to exclude data where parameters would be directly affected by a non–gonadotropin combination therapy, such as for serum testosterone concentrations in a patient receiving additional exogenous testosterone. Data heterogeneity and missing data were common across all studies, and reporting bias further limits our conclusions. Despite direct enquiries to the corresponding author, we were unable to obtain individual patient data from 3 studies (23, 24, 31), the data from which therefore could not be included in our data synthesis. The data synthesis was performed on observational data from patient groups that received different treatment regimens, with potential for confounding bias. Finally, we included only studies published in or translated into English.

## Conclusions

This systematic review provides robust evidence to support the efficacy of gonadotropin therapy in stimulating clinical and biochemical outcomes of mini-puberty in male infants with CHH. An increase was noted in testicular volume, SPL, resolution of cryptorchidism, and markers of Sertoli cell function. Therapies were well tolerated with good safety profiles. We identified substantial heterogeneity within and between all studies, regarding the choice, dose, and duration of treatment. This, and conclusions drawn, are limited further by a small sample size. Despite this, combined gonadotropin therapy in infancy holds promise both for immediate and potential long-term improvements in fertility and quality of life. Moreover, it is vital to raise awareness and emphasize the importance of an early diagnosis for patients with CHH. This enables therapeutic interventions to avoid or ameliorate long-term physical and psychosocial sequelae, as well as appropriate support for children and families.

## Data Availability

The data that support the findings of this study are available from the corresponding author on reasonable request.

## Abbreviations

AMH: antimüllerian hormone
CHH: congenital hypogonadotropic hypogonadism
FSH: follicle-stimulating hormone
GnRH: gonadotropin-releasing hormone
hCG: human chorionic gonadotropin
HH: hypogonadotropic hypogonadism
hMG: human menopausal gonadotropin
HPG: hypothalamic-pituitary-gonadal
IHH: idiopathic hypogonadotropic hypogonadism
IQR: interquartile range
LH: luteinizing hormone
rhFSH: recombinant human follicle-stimulating hormone
rhLH: recombinant human luteinizing hormone
ROBINS-I: Risk of Bias for Non-Randomized Studies of Interventions
SPL: stretched penile length
